# Value of the New Elastography Technique using Acoustic Radiation Force Impulse in Differentiation between Hashimoto’s Thyroiditis and Graves’ Disease

**DOI:** 10.25259/JCIS-22-2019

**Published:** 2019-05-24

**Authors:** Mohamed Mohamed Hefeda

**Affiliations:** Department of Radiodiagnosis, Faculty of Medicine, Tanta University, Gharbia, Egypt.

**Keywords:** Elastography, Hashimoto’s thyroiditis, Shear wave

## Abstract

**Background and Aim:**

This study was performed to evaluate the role and accuracy of shear wave elastography in the differentiation between Graves’ disease (GD) and Hashimoto’s thyroiditis (HT), in comparison with the B-mode ultrasound and color Doppler ultrasound.

**Materials and Methods:**

This study was non-randomized prospective study. The study included 30 patients with GD, 65 patients with HT, and 35 patients with normal thyroid glands. Assessment of ultrasonographic criteria, color Doppler flow pattern, and shear patterns differed significantly between the control group and the group of diffuse thyroid disease (*P* < 0.001). The most specific sign for diagnosing HT was nodularity (97.87%), and the most sensitive sign was coarse echotexture (81.54%). Color Doppler flow (CDF) showed sensitivity of 91.8%, specificity of 56.92%, positive predictive value of 52.54%, negative predictive value of 92.5%, and diagnostic accuracy of 68.69% in the differentiation between GD and HT. The mean shear wave velocity (SWV) was 2.61 ± 0.32 m/s in the GD group (range: 2.1–3.21 m/s), 2.85 ± 0.52 m/s in the HT group (range: 2.31–3.82 m/s), and 1.75 ± 0.37 m/s in the control group (range: 1.24–2.36 m/s). The mean SWVs in the GD and HT groups were significantly higher than that in the control group (*P* < 0.001). The mean SWV in the HT group was higher than that in the GD group (*P* = 0.03).

**Conclusion:**

Quantitative and qualitative SWE is useful for diagnosing diffuse thyroid disease and evaluating the degree of fibrosis in autoimmune thyroiditis. However, acoustic radiation force impulse techniques cannot differentiate between HT and GD reliably.

## INTRODUCTION

Both Graves’ disease (GD) and Hashimoto’s thyroiditis (HT) are autoimmune diseases of thyroid gland.^[1]^ GD is a genetic disorder and is the most common cause of hyperthyroidism and characterized by circulating thyroid-stimulating hormone receptor autoantibodies with thyroid-stimulating activity.^[2]^ On the other hand, HT is the most common cause of hypothyroidism.^[3,4]^ Pathologically, the disease is characterized by marked lymphocytic infiltration of the thyroid.^[5]^

Differentiation of GD from HT may sometimes be difficult on the basis of clinical and laboratory findings.^[1]^. Ultrasonography is a well-established technique in the diagnosis of thyroid diseases.^[6]^ However, recently, Pishdad *et al.*^[7]^ reported low sensitivity of sonography in differentiation between GD and HT and he concluded that ultrasound alone cannot differentiate between the two diseases due to marked overlapping in sonographic picture.

It was reported in the literature that increased arterial flow in the thyroid was not only seen in GD with hyperthyroidism but was also seen in HT with hypothyroidism.^[8]^ Thyroid gland hypervascularity is seen whatever the functional state and the level of thyroid hormones.^[9-12]^ Hence, color Doppler ultrasound may fail to differentiate between the two diseases with confidence.

Elastography is a relatively new technique measuring tissue stiffness and elasticity.^[13,14]^ Strain elastography and shear wave elastography (SWE) are the two basic types of elastography. Strain elastography uses external force for the prediction of tissue stiffness.^[15]^ SWE utilizes acoustic radiation force impulse (ARFI) imaging to measure the shear wave velocity (SWV) of the target area, which is considered quantitative operator independent. The SWV is calculated bythe production of small (1–10 mm) localized tissue displacements using short duration acoustic pulse; this tissue displacement results in shear wave dissemination, which is tracked and measured as SWV (m/s).^[16]^ Virtual touch tissue imaging quantification (VTIQ) (VTQ) is the name given to the new technique that quantifies tissue elasticity by calculating the SWV.^[17]^

In the current study, we evaluated the role and accuracy of SWE in the differentiation between GD and HT, in comparison with the B-mode ultrasound and color Doppler ultrasound.

## MATERIALS AND METHODS

### Patients

This study was non-randomized prospective study that was conducted between October 2017 and November 2018. The study included 30 patients with GD, 65 patients with HT, and 35 patients with normal thyroid glands. Informed consent was obtained from all patients.

#### Ultrasound machine

All patients underwent a sonographic examination of the thyroid gland using a Siemens ACUSON S3000 diagnostic ultrasound system (Siemens Medical Solutions, Erlangen, Germany). A Siemens 9L4 linear probe with a frequency range of 4–9 MHz was used in conventional ultrasonographic, color Doppler, and elastographic examinations.

#### Inclusion and exclusion criteria

The inclusion criteria for patients in the control group included normal sonographic appearance of the thyroid gland, normal serum levels of thyroid-stimulating hormone, free triiodothyronine, and free thyroxine. HT group was based on clinical findings, elevated levels of thyroglobulin antibodies, and/or antithyroperoxidase antibodies. The diagnosis of GD was based on clinical findings with high serum thyroxine and triiodothyronine. Excluded from the study patients with unsure diagnosis (either Hashimoto’s disease or GD), previous thyroid surgery, exposure to radioiodine therapy or radiation and patients with thyroid nodules either palpable clinically or diagnosed sonographically.

### Grayscale ultrasound

The sonographic features examined during the study were as follows: (1) the echogenicity included isoechoic, hypoechoic, markedly hypoechoic, and hyperechoic patterns. The strap muscles and submandibular glands were used as a reference for the determination of echogenicity; (2) echotexture: fine or coarse (heterogeneous); (3) presence of micronodularity (defined as numerous hypoechoic nodules <5 mm); (4) the presence of linear echogenic bands (echogenic septations); and (5) the anterior–posterior diameter of the thyroid on a longitudinal scan, which was divided into three categories: a normal range from 1 cm to 2 cm, small <1 cm, and large >2 cm.

### Color doppler ultrasound

Color Doppler flow images were obtained in the transverse and longitudinal planes and color gain was adjusted to a level not associated with troublesome artifacts with the probe repetition frequency at 750 Hz. The color flow Doppler (CFD) sonography patterns were as follows: Pattern 0: absent intraparenchymal vascularity or minimal spots; Pattern I: spotty uneven distribution; Pattern II: mild increase of CFD signal with patchy distribution; and Pattern III: markedly increased color flow signals with diffuse homogeneous distribution (including thyroid inferno).^[18]^

### Shear wave elastography

During the B-mode ultrasonography examination, the elastographic mode was switched on. Only light pressure was exerted by the probe on the tissues, and the patients were asked to stop breathing and swallowing. VTQ was performed on B-mode ultrasonographic images with a fixed region of interest (ROI) measuring 5 mm × 6 mm. The ROI box was placed completely within the thyroid parenchyma. The SWV was then measured. The SWV is assumed to be proportional to the square root of the tissue elasticity. Three measurements were performed on the right thyroid lobe and three on the left thyroid lobe. The average of the six values was calculated. The range of SWV was 0–9 m/s. Furthermore, VTIQ map is used for creating shear wave image and subsequent tissue quantification in one display. We followed the four-point system which is as follows: Score 1, the ROI is entirely bright; Score 2, the ROI is mostly bright, with some dark areas; Score 3, the ROI is mostly dark, with some bright areas; and Score 4, the ROI is entirely dark.^[19]^

### Statistical analysis

Statistical analysis was performed using SPSS software, version 22 (IBM Corporation, NY, USA). The differences of SWV between the different groups were compared; *P* < 0.05 was considered to be statistically significant. All measurements were expressed as the mean value ± standard deviation. The sensitivity, specificity, positive predict value, negative predict value, and diagnostic accuracy were calculated with Chi-square test.

## RESULTS

In total, 130 patients (18 male, 112 female; mean age: 37 years; age range: 18–52 years) were included in the study. Sixty-five patients with HT (mean age, 41 years), 35 patients with GD (mean age, 33 years), and 30 healthy controls (median age, 29 years) were examined. The groups were similar in terms of age and sex. No significant difference in the mean age between the different groups (*P* = 0.61). The GD group included 22 patients was non treated and 13 patients received previous treatment. The HT group was divided into early disease (18 patients) and chronic disease (47 patients) (based on history, clinical examination, and laboratory findings).

### Grayscale ultrasound

The grayscale patterns differed significantly between the control group and the group of diffuse thyroid disease (*P* < 0.001). However, there was marked overlap in the sonographic features between the HT and GD groups. Homogeneous hypoechogenicity was seen in 43.1% of Hashimoto’s cases, 74.1% of GD cases, and 23.3% of control group [Table 1], while marked hypoechogenicity was observed in 34% of patients with HT. Marked hypoechogenicity as a sign of HT had sensitivity of 55.74%,specificity of 79.50%, positive predictive value (PPV) of 80.95%, negative predictive value (NPV) of 53.45%, and a diagnostic accuracy of 65%.

**Table 1:** Grayscale ultrasound findings in studied groups (number and percentage).

Ultrasound	Control group *n*=30 (%)	HT *n*=65 (%)	GD *n*=35 (%)	*P* value between control group and the two other groups	*P* value between HT and GD groups
Echogenicty				<0.001	0.76
Isoechoic	22 (73.3)	3 (4.6)	1 (2.9)		
Hypoechoic	7 (23.3)	28 (43.1)	26 (74.3)		
Markedly hypoechoic	-	34 (52.3)	8 (22.9)		
Hyperechoic	1 (3.3)	-	-		
Echotecture				<0.001	<0.05
Fine	30 (100)	12 (18.5)	18 (51.4)		
Coarse	-	53 (81.5)	17 (48.6)		
Size				<0.001	0.15
<1 cm	2 (6.7)	7 (10.8)	-		
1–2 cm	27 (90)	21 (32.3)	9 (25.7)		
>2 cm	1 (3.3)	37 (56.9)	26 (74.3)		
Echogenic septations				<0.001	0.78
Yes	2 (6.7)	24 (36.9)	12 (34.2)		
No	28 (93.3)	41 (63.1)	23 (65.7)		
Nodularity				<0.01	<0.03
Yes	-	19 (29.2)	1 (2.9)		
No	30 (100)	46 (70.8)	34 (97.1)		

HT: Hashimoto’s thyroiditis, GD: Graves’ disease

Gland size, presence of echogenic septations, and echogenicity showed no significant difference between the HT and GD groups. While nodularity was observed in 29.2% and 2.9% of Hashimoto’s groups and GD s respectively (*P* = 0.03), also coarse texture observed in 81.5% of HT and 48.6% of GD patients (*P* < 0.05). The most specific sign for diagnosing HT was nodularity (97.87%), and the most sensitive sign was coarse echotexture (81.54%) [Tables 1 and 2].

**Table 2:** The diagnostic performance of different grayscale parameters in differentiation between HT and GD.

Ultrasound	Sensitivity (%)	Specificity (%)	PPV (%)	NPV (%)	Accuracy (%)
Echogenicty markedly hypoechoic echopattern as sign of HT	55.74	79.49	80.95	53.45	65
Coarse echotexture as a sign of HT	81.54	51.43	75.71	60.00	71
Echogenic septations as a sign of HT	63.08	34.29	64.00	33.33	53
Nodularity as a sign of HT	35.83	97.87	95.00	57.50	65

GD: Graves’ disease, SWV: Shear wave velocity, HT: Hashimoto’s thyroiditis

### Color doppler ultrasound

The color flow pattern in the control group limited to Patterns 0 and I. In HT, 28 patients (43.1%) showed hypervascularity. On the other hand, the Pattern III was seen only in patients with GD, 25 patients (71.4%). CFD showed a sensitivity of 91.8%, specificity of 56.92%, PPV of 52.54%, NPV of 92.5%, and diagnostic accuracy of 68.69% in the differentiation between GD and HT. Furthermore, patients with diffuse thyroid disease had hypervascularity than control group (*P* < 0.01). Hypervascular patterns found in patients with GD than patients with HT [Tables 3 and 4].

**Table 3:** Color Doppler patterns in different groups.

Doppler pattern	Control group *n*=30 (%)	HT *n*=65 (%)	GD *n*=35 (%)	*P* value between control group and the two other groups	*P* value between HT and GD groups
Pattern 0	12 (40)	13 (20)	-	<0.01	<0.05
Pattern I	18 (60)	24 (36.9)	3 (8.6)		
Pattern II	-	28 (43.1)	6 (17.1)		
Pattern III	-	-	25 (71.4)		

HT: Hashimoto’s thyroiditis, GD: Graves’ disease

**Table 4:** Diagnostic performance of hypervascularity as a sign of GD.

	Sensitivity	Specificity	PPV	NPV	Accuracy
Hypervascularity Patterns II and III as a sign of GD	91.8%	56.92%	52.54	92.5	68.69%

GD: Graves’ disease, PPV: Positive predictive value, NPV: Negative predictive value

### Shear wave elastography

**Figure 1:**
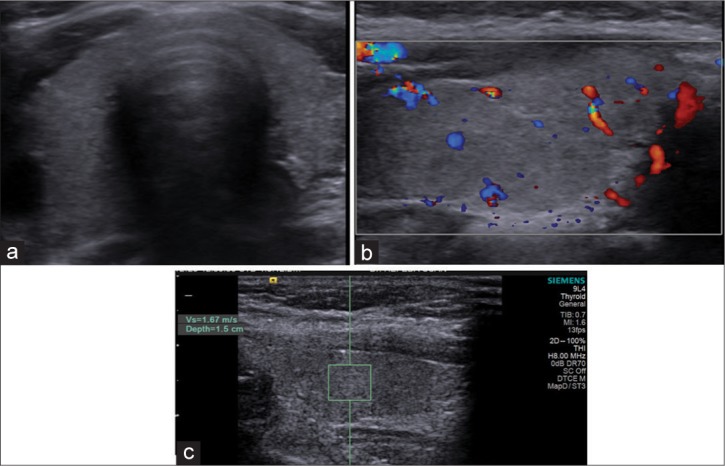
Patient with normal thyroid gland: (a) Grayscale ultrasound showing smooth homogenous isoechoic echopattern. (b) Color Doppler: Type I pattern. (c) Shear wave velocity 1.67 m/s (depth 1.5 cm).

**Figure 2:**
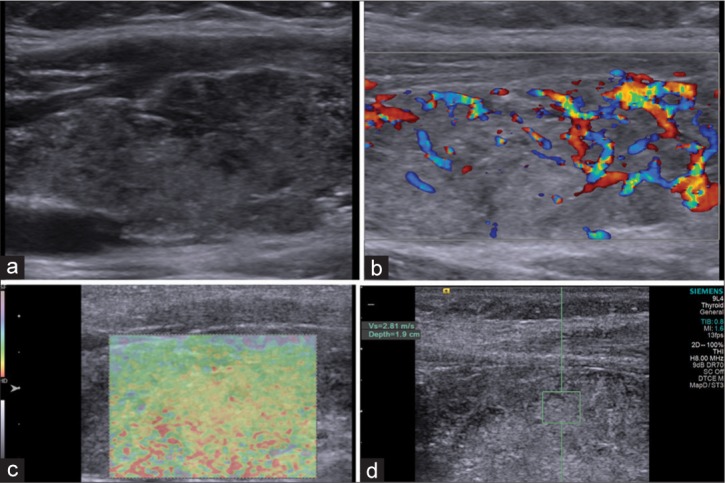
Patient with Hashimoto’s thyroiditis: (a) Grayscale ultrasound showing hypoechoic heterogeneous echopattern. (b) Color Doppler: Type II pattern. (c) Elstogram reveals type III pattern. (d) Shear wave velocity 2.81 m/s (depth 1.9 cm).

**Figure 3:**
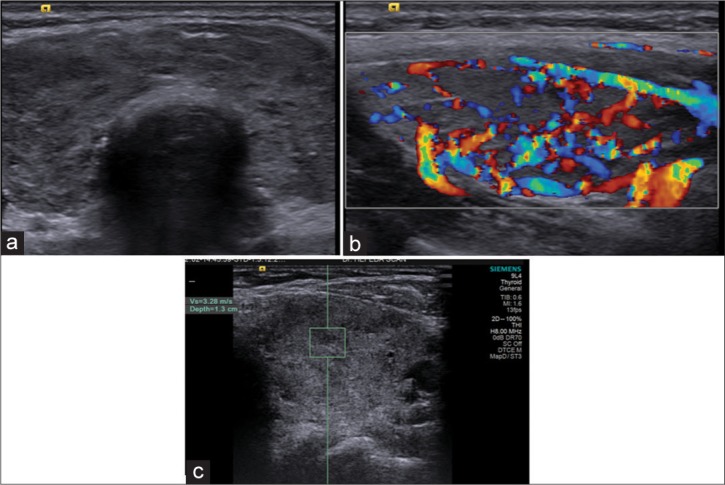
Patient with Hashimoto’s thyroiditis: (a) Grayscale ultrasound showing hypoechoic heterogeneous echopattern with miconodularity. (b) Color Doppler: Hypervascularity with Type II pattern. (c) Shear wave velocity 3.28 m/s (depth 1.3 cm).

**Figure 4:**
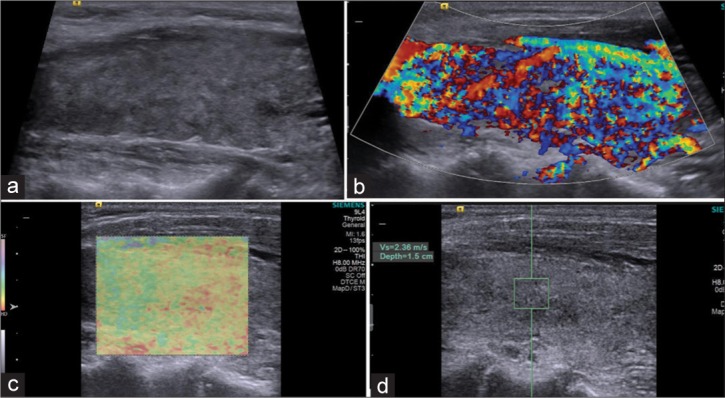
Patient with Graves’ disease: (a) Grayscale ultrasound showing hypoechoic echopattern. (b) Color Doppler: Type III pattern (thyroid inferno). (c) Elstogram reveals type II pattern. (d) Shear wave velocity 2.36 m/s (depth 1.5 cm).

**Figure 5:**
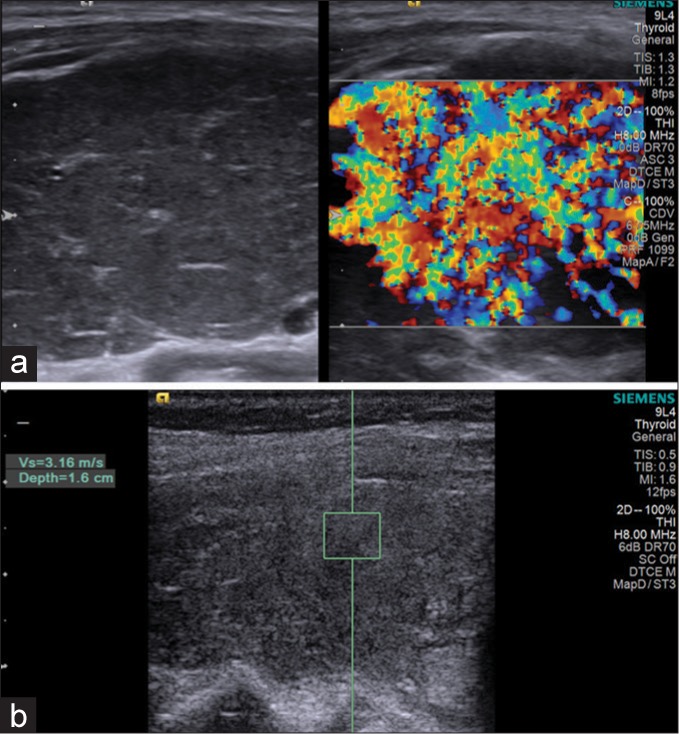
Patient with Graves’ disease: (a): Hypoechoic parenchyma with marked hypervascularity. (b): Shear wave velocity = 3.16 m/s (depth = 1.6 cm).

The mean SWV was 2.61 ± 0.32 m/s in the GD group (range: 2.1–3.21 m/s), 2.85 ± 0.52 m/s in the HT group (range: 2.31–3.82 m/s), and 1.75 ± 0.37 m/s in the control group (range: 1.24–2.36 m/s) [Tables 5 and 6]. The mean SWVs in the GD and HT groups were significantly higher than that in the control group (*P* < 0.001). The mean SWV in the HT group was higher than that in the GD group (*P* = 0.03) [Figures 1-5].

**Table 5:** Results of shear wave elastography.

	Control	HT	GD	*P* value between normal and the two groups	*P* value between HT and GD
SWV (m/s)				<0.001	0.03
Mean	1.75±0.37	2.85±0.52	2.61±0.32		
Range	1.24–2.36	2.3–3.8	2.1–3.21		
Elastograpram				0.15	0.025
Pattern I	19 (63.3%)	9 (13.8%)	9 (25.7%)		
Pattern II	11 (36.7%)	25 (38.4%)	16 (45.7%)		
Pattern III		31 (47.7%)	10 (25.6%)		
Pattern IV			-		

HT: Hashimoto’s thyroiditis, GD: Graves’ disease

**Table 6:** Shear wave velocity between different groups subgroups (m/s).

SWV (m/s)	HT		GD	
	Early HT (*n*=18)	Chronic HT (*n*=47)	Non-treated (*n*=22)	Treated (*n*=13)
Mean	2.31±0.24	2.63±0.56	2.31±0.23	2.49±0.41
Range	2.3–2.7	2.34–3.8	2.1–2.6	2.3–3.21

Statistically significant difference between chronic HT and early HT (*P*<0.01) and non-treated GD (*P*<0.01), and no significant difference between treated and untreated GD and early HT (*P*=0.72 and 0.34, respectively). There was a significant difference between treated and non-treated GDs (*P*=0.01). HT: Hashimoto’s thyroiditis, GD: Graves’ disease, SWV: Shear wave velocity

In the GD group, the mean SWV of patients who were and were not undergoing antithyroid therapy was 2.31 ± 23 m/s and 2.49 ± 41 m/s, respectively (*P* < 0.01). Patients who were undergoing antithyroid therapy showed a significantly higher SWV than patients who were not undergoing antithyroid therapy.

In the HT group, the mean SWV of patients with early disease and chronic disease was 2.31 ± 0.24 m/s and 2.63 ± 0.56 m/s, respectively. The difference was statistically significant (*P* < 0.01).

There was no significant difference between treated and untreated GD and early HT (*P* = 0.72 and 0.34, respectively).

Real-time elastography was studied in the current study. No lobes showed Pattern IV. Patter III was seen in 47.7% of patients with HT and 25.6% of patients with GD (*P* = 0.025).

Furthermore, we tried to correlate the SWV and the different sonographic and color Doppler parameters [Table 7]. High velocities were seen in lobes with coarse echotexture, nodularity, and fine septations (*P* < 0.05, < 0.01, and < 0.01, respectively), which would probably correlate with the degree of fibrosis.

**Table 7:** Correlation between sonogaphic findings and SWV.

Ultrasound	SWV	*P* value between HT and GD groups
Echogenicty		
Iso/mildly hypoechoic	2.42±0.72	0.43
Markedly hypoechoic	2.62±0.61	
Echotecture		<0.05
Fine	1.98±0.52	
Coarse	2.82±0.61	
Size		0.38
<1 cm	2.32±0.22	
1–2 cm	2.02±0.41	
>2 cm	2.58±0.52	
Echogenic septations		<0.01
Yes	2.87±0.32	
No	2.31±0.51	
Nodularity		<0.01
Yes	2.98±0.47	
No	2.62±0.61	
Color flow pattern		0.34
Hypervascular (Patterns II and III)	2.68±0.41	
Non hypervascular (Patterns 0 and I)	2.52±0.23	

GD: Graves’ disease, SWV: Shear wave velocity, HT: Hashimoto’s thyroiditis

## DISCUSSION

This study confirms that the SWV of the thyroid glands with autoimmune thyroiditis (both GD and HT) is higher than normal glands, and the SWV increases with the degree of fibrosis. Although the SWV differs between the GD and the HT, there is overlap in velocities between the two groups.

The ARFI is an emerging technique providing information about tissue stiffness. It contains two modes, VTI and VTQ. They provide the information about tissue elasticity qualitatively and quantitatively, respectively.^[20]^

Stiffness or elasticity is an important physical parameter of biological tissue; in GD, elasticity is increased due to diffuse hypertrophy and hyperplasia of follicular cells, colloid depletion, and lymphoid infiltration as well as hypervascularity.^[21]^ On the other hand, lymphocytic infiltration and fibrosis occurring in the thyroid gland of HT increase the stiffness of the thyroid tissue.^[15]^ Correspondingly, in the current study, the mean SWVs in the GD and HT groups were significantly higher than that in the control group (*P* < 0.001); this objective index might make the sonographer more confident in making developmental trauma disorder diagnosis. Nevertheless, our findings showed that although there was statistically significant difference between the SWVs in both HT and GD, there was marked overlapping between the two groups, making differentiation between the two groups unreliable.

In the current study, the mean SWV was 2.61 ± 0.32 m/s in the GD group (range: 2.1–3.21 m/s), 2.85 ± 0.52 m/s in the HT group (range: 2.31–3.82 m/s), and 1.75 ± 0.37 m/s in the control group (range: 1.24–2.36 m/s). The higher elasticity in diffuse thyroid disease is in agreement with previous studies. Two studies using strain ratios done by Ruchala *et al.*^[22]^ and Menzilcioglu *et al.*^[23]^ found higher strain ratios in diffuse thyroid disease than control group and were compatible with our study. Hekimoglu *et al.*^[14]^ found SWV in control group (1.63 ± 0.12 m/s) which is close to our results (1.75 ± 0.37 m/s) and they found significantly higher velocity in the group of chronic autoimmune thyroiditis than the control group. Furthermore, Fukuhara *et al.*^[24]^ compared SWVs between patients with CAT and a healthy control group using SWE by ARFI.^[11]^ They found that the mean SWV in patients with CAT was 2.47 ± 0.57 m/s, which was significantly higher than that in the control group (1.59 ± 0.41 m/s). All these studies were in agreement with the current study that the SWV of the patient group (HT + GD) is significantly higher than that of the control group.

The higher SWV in HT than GD is not in agreement with two previous reports. Sporea *et al.*^[25]^ compared the SWV among 29 patients with GD, 22 patients with CAT, and 23 control subjects and found a significantly higher SWV in the GD than in the CAT group (2.82 ± 0.47 vs. 2.49 ± 0.48 m/s, respectively). Furthermore, Rahatli *et al.*^[26]^ in a study included 30 patients with HT and 22 patients with GD found patients with GD had significantly higher shear wave velocities than those with HT (*P* < 0.001). The difference between the results of current study and these two studies may be due to difference in number of the patients, and the fact that most of patients with HT included in the study was in chronic stage. On the other hand, our study is in agreement with previous study by Du *et al.*,^[27]^ who in a study included 74 patients with diffuse thyroid disease and 30 normal controls found higher SWV in HT than GD groups (2.72 ± 1.52 m/s, vs. 2.14 ± 0.31 m/s), but they reported the difference as statistically insignificant.

Liu *et al.*^[28]^ recently reported that the SWV is not suitable for differentiation between chronic thyroiditis and GD, and this assumption was confirmed by our study due to marked overlapping between the two groups. In the current study, the SWV was higher in treated GD than non-treated GD and there was a significant difference between chronic HT and early HT. The cause of the marked overlapping observed in our study probably related to the presence of cases with non-treated GD and early HT, both of them have similar SWV.

Kim *et al.*^[29]^ in a prospective study found that real-time thyroid US is useful in differentiating diffuse thyroid disease from normal thyroid parenchyma. In the current study, grayscale ultrasound proved accurate in differentiation between normal thyroid tissue and patients with HT or GD. However, regarding differentiation between HT and GD, there was a significant overlap between the sonographic pictures, with only coarse texture and nodularity were significantly more common in HT than GD. In the current study, we studied each ultrasound sign separately for the differentiation between HT and GD and found that most specific sign for diagnosing HT was nodularity (97.87%) and the most sensitive sign was coarse echotexture (81.54%). No sign had high sensitivity and specificity. These results are similar to the study by Pishdad *et al.*^[7]^ who reported that sonography had a high specificity (90.6%) but low sensitivity (47.1% and 45.2%) in the diagnosis and differentiation of GD and HT. Furthermore, Tabur *et al.* found that sonography has high specificity (90%) and low sensitivity (35%) in the diagnosis of thyroiditis.^[30]^ Furthermore, in the current study, coarse echotexture as a sign of HT had a sensitivity of 81.54%, a result similar to those of Patel *et al.*, who found that diffuse heterogeneity had a sensitivity of 88.2% in the diagnosis of HT.^[31]^

Furthermore, we tried to correlate the SWV and the different sonographic signs. High velocities were seen in lobes with coarse echotexture, nodularity, and fine septations (*P* < 0.05, < 0.01, and < 0.01, respectively). The presence of these signs may correlate with the degree of fibrosis. Our results are similar to those of Kandemirli *et al.*^[32]^ who in a study included that 85 patients found high elasticity values in that thyroid glands with marked hypoechoic, septations, and pseudonodular appearance.

In the current study, the color Doppler ultrasound showed a sensitivity of 91.8% and a specificity of 56.92% in the differentiation between HT and GD. The low specificity is due to the fact that 28 patients (43.1%) with HT showed hypervascularity (Pattern II), but the Pattern III was seen only in patients with GD. Increased vascularity in HT was previously reported.^[33,34]^ Ceylan *et al.*^[35]^ reported hypervascularity in 85% of their patients with HT. The presence of HT with hypervascularity leads to the low specificity of CDF in differentiation between HT and GD.

A limitation of this study is that biopsy and/or pathological specimens were not available for all patients; we relied mainly on the clinical and laboratory findings. The HT group and GD were unequal, with a larger number of early and chronic HT. No inter- and intra-observer data were tried. The last to mention is that only the GD and HT patients were involved. Other types of diffuse thyroid disease were not included in the study.

## CONCLUSION

Quantitative and qualitative SWE is useful for diagnosing diffuse thyroid disease and evaluate the degree of fibrosis in autoimmune thyroiditis. However, ARFI techniques cannot alone differentiate between HT and GD reliably, but the addition of the technique to the conventional ultrasound and color Doppler ultrasound may facilitate the differentiation between the two diseases.
